# bFGF-mediated pluripotency maintenance in human induced pluripotent stem cells is associated with NRAS-MAPK signaling

**DOI:** 10.1186/s12964-018-0307-1

**Published:** 2018-12-05

**Authors:** Fereshteh Haghighi, Julia Dahlmann, Saeideh Nakhaei-Rad, Alexander Lang, Ingo Kutschka, Martin Zenker, George Kensah, Roland P. Piekorz, Mohammad Reza Ahmadian

**Affiliations:** 1 Institute of Biochemistry and Molecular Biology II, Medical Faculty of the Heinrich Heine University, Düsseldorf, Germany; 2 Department of Thoracic and Cardiovascular Surgery, University of Göttingen, Göttingen, Germany; 3 Department of Cardiothoracic Surgery, University Clinic, Otto von Guericke-University, Magdeburg, Germany; 4 Institute of Human Genetics, Otto von Guericke-University, Magdeburg, Germany; 5 Present address: Department of Urology, Medical Faculty of Heinrich Heine University, Düsseldorf, Germany

**Keywords:** bFGF, Differentiation, Induced pluripotent stem cells, MAPK, RAS, PI3K, Pluripotency

## Abstract

**Background:**

Human pluripotent stem cells (PSCs) open new windows for basic research and regenerative medicine due to their remarkable properties, i.e. their ability to self-renew indefinitely and being pluripotent. There are different, conflicting data related to the role of basic fibroblast growth factor (bFGF) in intracellular signal transduction and the regulation of pluripotency of PSCs. Here, we investigated the effect of bFGF and its downstream pathways in pluripotent vs. differentiated human induced (hi) PSCs.

**Methods:**

bFGF downstream signaling pathways were investigated in long-term culture of hiPSCs from pluripotent to differentiated state (withdrawing bFGF) using immunoblotting, immunocytochemistry and qPCR. Subcellular distribution of signaling components were investigated by simple fractionation and immunoblotting upon bFGF stimulation. Finally, RAS activity and RAS isoforms were studied using RAS assays both after short- and long-term culture in response to bFGF stimulation.

**Results:**

Our results revealed that hiPSCs were differentiated into the ectoderm lineage upon withdrawing bFGF as an essential pluripotency mediator. Pluripotency markers OCT4, SOX2 and NANOG were downregulated, following a drastic decrease in MAPK pathway activity levels. Notably, a remarkable increase in phosphorylation levels of p38 and JAK/STAT3 was observed in differentiated hiPSCs, while the PI3K/AKT and JNK pathways remained active during differentiation. Our data further indicate that among the RAS paralogs, NRAS predominantly activates the MAPK pathway in hiPSCs.

**Conclusion:**

Collectively, the MAPK pathway appears to be the prime signaling pathway downstream of bFGF for maintaining pluripotency in hiPSCs and among the MAPK pathways, the activity of NRAS-RAF-MEK-ERK is decreased during differentiation, whereas p38 is activated and JNK remains constant.

**Electronic supplementary material:**

The online version of this article (10.1186/s12964-018-0307-1) contains supplementary material, which is available to authorized users.

## Background

Embryonic stem cells (ESCs) are derived from the inner cell mass of human blastocyst [1], and represent promising tools in tissue engineering and cell therapy [2, 3]. What makes these pluripotent cells so valuable in developmental biology and regenerative medicine is their ability to differentiate into cell-types of different lineages both in vivo and in vitro. In order to realize the potential of ESCs in clinical applications, it is crucial to address fundamental questions regarding their molecular nature of pluripotency and the underlying intracellular signaling pathways which maintain the characteristics of these cells.

Various signaling pathways, including basic fibroblast growth factor (bFGF/FGF2), TGF-β/activin, WNT, EGFR family, insulin/IGF, PDGF, neurotrophin, integrin and NOTCH, participate in maintaining pluripotency in hESCs [4–12]. Among the 22 FGF ligands, it is widely accepted that hESCs require exogenous bFGF to sustain self-renewal and the capacity to differentiate into a large number of somatic cell types [13]. bFGF maintains pluripotency either directly under feeder-free conditions supplemented with activin A [14] or indirectly by stimulating irradiated mouse embryonic fibroblasts (iMEFs) to secrete activin A and other growth factors and cytokines necessary for hESCs pluripotency [15–17]. Therefore, among many growth factors and cytokines in maintaining pluripotency of hESCs and hiPSCs, bFGF was selected for monitoring downstream signaling pathways.

The stimulation of the bFGF receptors result in activation of various signaling pathways, including MAPK, PI3K/AKT and JAK/STAT [18]. The former two pathways are activated via RAS proteins, which control essential cellular processes, such as proliferation, differentiation, apoptosis, adhesion and migration and thus embryogenesis and normal development [19–21]. However, not much is known about the role of RAS proteins in regulating pluripotency or differentiation of hPSCs, especially hiPSCs. It has been shown that RAS proteins regulate the transition from naïve to primed ESCs in mice [22] and RAS nullyzygosity reduces the proliferation of mouse (m) ESCs and prohibits their differentiation [23].

In this study, we explored and expanded the molecular mechanism involved in the transition from pluripotency to differentiation with the focus on bFGF signaling in hiPSCs. We showed that, pluripotency markers were downregulated in hiPSCs following bFGF withdrawal and differentiated toward the ectoderm lineage. The MAPK pathway activity was significantly decreased, but interestingly no relevant changes were found in the activation of AKT or its downstream targets such as S6 kinase (S6K), FOXO-1, and JNK pathway. Investigating other signaling pathways downstream of bFGF revealed the activation of JAK/STAT3 and p38 as a result of differentiation. Moreover, we identified NRAS among the RAS paralogs as the likely link between bFGF receptor and the MAPK pathway that maintains hiPSCs undifferentiated.

## Methods

### Cell culture

Two clones of hiPSCs were generated by electroporating human foreskin fibroblasts (HFF, purchased from ATCC) with non-integrating episomal reprogramming vectors obtained from Addgene (pCE-hSK #41814, pCE-hOct3/4 #41813, pCE-hUL #41855, pCE-mp53DD #41856, pCXB-EBNA1 #41857) as previously described [24]. After 3–4 weeks, emerging hiPSC colonies were manually dissected under microscopic control and plated individually on mitotically inactivated (30 Gy gamma irradiation) iMEFs. Established clones were maintained as colonies on feeder layers in medium comprised of Dulbecco’s Modified Eagle’s Medium/Ham’s F12 + GlutaMAX (DMEM/F12) (ThermoFisher, 31,331–028) supplemented with 20% knockout serum replacement (KSR) (ThermoFisher, 10,828,028), 1% non-essential amino acids (NEAA) (ThermoFisher, 11,140,035), 0.1 mM 2-mercaptoethanol (Millipore, ES-007.E), 25 ng/mL bFGF (Peprotech, 100-18B) and 50 units of penicillin/streptomycin (Genaxxon Bioscience, M3140.0100). The medium was changed every second day and colonies were passaged once per week using 0.4% (*w/v*) collagenase IV (ThermoFisher, 17,104,019). To initiate feeder-free cultures, almost confluent colony cultures were dissociated using Accutase (ThermoFisher, A1110501) and seeded onto Geltrex coated dishes (ThermoFisher, A14132–02, 1:400) at a seeding density of 2 × 10^5^ cells/cm^2^ with iMEF conditioned medium (CM, see below) supplemented with 100 ng/mL of bFGF and 10 μM Y27632 ROCK inhibitor (Selleckchem, S1049). After approximately 24 h, medium was exchanged with CM plus 100 ng/mL bFGF but without Y27632. Medium was replaced daily and confluent hiPSC monolayers were passaged every 3–4 days in the same manner using Accutase with seeding densities of 5 × 10^4^ cells/cm^2^. The CM was prepared as previously described [13]. Briefly, iMEFs were seeded at the density of 6 × 10^4^ cells/cm^2^ on precoated dishes with 1% gelatin (Sigma, G9391). One day after seeding, iMEFs were washed with PBS without calcium and magnesium (ThermoFisher, 10,010–015) and the medium was exchanged with DMEM/F12, 15% KSR, 1% NEAA, 0.1 mM 2-mercaptoethanol and 5 ng/mL bFGF. CM was replaced daily and collected for 7 days, filtered and aliquoted. HeLa and HFF cells were cultured in DMEM (ThermoFisher, 11,965,092), whereas NT2 cells were cultured in McCoy’s media (ThermoFisher, 16,600,082), all supplemented with 10% FBS (ThermoFisher, 10,270–106) and 50 units of penicillin/streptomycin. Cell pellets from astrocytes were a gift from Dr. Boris Görg from the Heinrich-Heine University Düsseldorf.

### Long-term stimulation

In order to investigate the effect of bFGF and its downstream pathways on hiPSCs, cells were cultured under four different conditions; 100 ng/ml bFGF (CM-100) as the standard condition that was added to the medium each day freshly, 5 ng/ml bFGF (CM-5) as a lower concentration of bFGF, the conditioned medium without any bFGF (CM-0) and non-conditioned medium (non-CM) (as negative control) that contains only DMEM/F12, 15% KSR, 1% NEAA and 0.1 mM β-mercaptoethanol. For preparing CM-0, iMEFs were supplemented with DMEM/F12, 15% KSR, 1% NEAA, 0.1 mM β-mercaptoethanol without any bFGF and the CM-0 was collected daily for 7 days. Undifferentiated hiPSCs were seeded on Geltrex coated dishes with a density of 5 × 10^4^ cells/cm^2^ with CM-100 supplemented with 10 μM Y27632. The day after, cells were washed with PBS^−/−^ and treated with CM-100, CM-5, CM-0 and non-CM and were kept in culture for 6 days. The medium was changed every day and at day 3, all cells were passaged by Accutase and seeded with the specific medium with the addition of 10 μM Y27632. Cells were harvested at the end of day 6 for RNA and protein level analysis.

### Short-term stimulation

hiPSCs were seeded at a density of 5 × 10^4^ cells/cm^2^ and cultured until they reached 70–80% confluency and then washed two times with PBS^−/−^ and incubated overnight (12 h) in DMEM/F12 deprived of bFGF and KSR. Cells were stimulated with 100 ng/ml bFGF for 15, 30, 60 and 120 min before preparation of cell lysates.

### Reverse transcriptase polymerase chain reaction

The total RNA was extracted by the RNeasy Plus kit (Qiagen, Germany) according to the manufacturer’s protocol. The quantity of the isolated RNA samples was analyzed by Nanodrop spectrophotometer. DNA-free™ DNA removal kit (Ambion, Life Technologies) was used to get rid of any possible contamination with genomic DNA. Complementary DNA (cDNA) was synthesized from DNase-treated RNA using ImProm-IITM reverse transcription system (Promega, Germany) and real-time PCR was performed using SYBR Green reagent (Life Technologies). *GAPDH* was used as an internal control. The 2^-Δct^ method was used for estimating the relative mRNA expression levels. Primer sequences are listed in Additional file 1: Table S1.

### Immunoblotting

Cell lysates were prepared using lysis buffer (50 mM Tris-HCl, pH 7.5, 100 mM NaCl, 2 mM MgCl_2_, 1% Igepal CA-630, 10% glycerol, 20 mM β-glycerolphosphate, 1 mM Na_3_VO_4_, EDTA-free protease inhibitor (Roche Applied Science)), and protein concentrations were measured by Bradford assay (Bio-Rad). Equal amount of total cell lysates (t-p38/p-p38; STAT3/p-STAT3; t-JNK/p-JNK; t-RSK/p-RSK1: 40 μg; the remaining proteins: 10 μg) were loaded on SDS-PAGE. After electrophoresis, proteins were transferred into nitrocellulose membrane and blocked for one hour in 5% nonfat dry milk (Merck)/TBST (Tris-buffered saline, 0.05% Tween 20). Then membranes were probed with primary antibody at 4 °C overnight and later stained for one hour at room temperature with both horseradish peroxidase (HRP)-conjugated secondary antibodies (1:5.000 dilution) and florescence secondary antibodies (1:10.000 dilution). Signals were visualized using ECL (enhanced chemiluminescence) reagent (GE Healthcare) and the Odyssey Fc Imaging System (LI-CORE Biosciences) respectively. The following antibodies were applied for immunoblotting: mouse anti-γ-tubulin (Sigma-Aldrich, T5326); mouse anti-OCT4 (Santa Cruz, sc-5279); rabbit anti-SOX2 (Invitrogen, PA1–16968); goat anti-NANOG (R&D systems, AF1997); mouse anti-SSEA4 (Millipore, MAB4304); mouse anti-α-SMA (DAKO, M0851); rabbit anti-GFAP (DAKO, Z0334); rabbit anti-MEK1/2 (#9126), rabbit anti-ERK1/2 (#9102), rabbit anti-RSK (#9355), rabbit anti-AKT (#9272), rabbit anti-p-MEK1/2 (S217/S221, #9154), rabbit anti-p-ERK1/2 (T202/T204, #9106), rabbit anti-p-p90RSK (T573, #9346), rabbit anti-p-AKT (S473, # 4060 and T308, #2965), rabbit anti-FOXO1 (#2880), rabbit anti-p-FOXO1 (S256, #9461), rabbit anti-S6 kinase (#2708), rabbit anti-p-p70 S6 kinase (T389, #9205), rabbit anti-p38 (#8690), rabbit anti-p-p38 (T180/Y182, #9211), rabbit anti-JNK (#9252), rabbit anti-p-JNK (T183/Y185, #9251), mouse anti-STAT3 (#9139S) and rabbit anti-p-STAT3 (Y705, #9145S) all from Cell Signaling.

### Flow cytometry

For flow cytometric analysis, single-cell suspensions were obtained with Accutase and cells were washed with ice-cold PBS^−/−^. Cells were fixed in 4% paraformaldehyde (PFA; Merck) for 10 min on ice and permeabilized with 90% ice-cold methanol for 15 min followed by a blocking step with 1.5% BSA and 2.5% goat or donkey serum diluted in PBS for 1 h at 4 °C. Cells were stained with primary antibodies overnight at 4 °C. Secondary antibodies, Alexa Fluor 488-conjugated goat anti-mouse IgG (A11029) and Alexa Fluor 488-conjugated donkey anti-rabbit IgG (A21206) from Invitrogen, were used at a dilution of 1:2000 for one hour at room temperature. Samples were analyzed with FACS Canto II (BD Pharmingen) and FlowJo Software (Treestar, Ashland, OR).

### Cell fractionation

Simple fractionation was performed as previously described [25]. Briefly, cells were washed with ice-cold PBS. 1 ml lysis buffer (without Igepal CA-630) was added to cells following centrifugation for 10 s, at 12000 rpm and 4 °C. The supernatant was removed and the pellet was resuspended in 900 μl ice-cold lysis buffer with 0.1% NP40 and was kept on ice for 2 min. 300 μl was taken as total cell lysate (TCL) and 100 μl of 4x Laemmli buffer was added to it. The rest of the supernatant was centrifuged and 300 μl was taken as cytosolic fraction (Cyt) following adding 100 μl of 4x Laemmli buffer. The remaining pellet was resuspended in 1 ml ice-cold lysis buffer with 0.1% NP40 and centrifuged again. The pellet was resuspended in 380 μl 1x Laemmli buffer and kept as nuclear fraction (Nuc). TCL and Nuc were sonicated at level 2 for 5 s and all fractions were boiled for 10 min at 95 °C. 20 μl from each fraction was loaded on SDS-PAGE. GAPDH (Cell signaling, #2118) and Na^+^/K^+^ ATPase (Sigma, A276) were used as cytosolic markers and histone H3 (Cell Signaling, #9715) and lamin B1 (Abcam, 16,048) were subjected as nuclear markers.

### Immunostaining

Cells were seeded on Geltrex-coated coverslips with the density of 5 × 10^4^ cells/cm^2^. Cells were washed with PBS containing magnesium/calcium two times and fixed with 4% PFA for 20 min at room temperature. To permeabilize cell membranes, cells were incubated in 0.25% Triton X-100/PBS for 5 min. Blocking was performed by using 3% BSA in PBS for one hour, room temperature. Incubation with primary antibodies was performed overnight at 4 °C following three times washing steps with PBS and then incubation with secondary antibodies Alexa Fluor 488-conjugated goat anti-mouse IgG, Alexa Fluor 488-conjugated donkey anti-rabbit IgG, Alexa Fluor 488-conjugated donkey anti-goat IgG (A11003) all from Invitrogen, used at a dilution of 1:500 for two hours at room temperature. Slides were washed three times and then stained with 4′,6-diamidino-2 phenylindole (DAPI) (Life Technologies) for five minutes and washed again for two times. ProLong Gold antifade was applied to mount coverslips. Confocal images were obtained using a LSM 510-Meta microscope (Zeiss, Jena, Germany).

### Pull-down assay

The RAS-binding domains (RBD) of effector proteins, including CRAF-RBD (a.a. 51–131) and PI3K-RBD (a.a. 127–314) were constructed as GST-fusions in pGEX-4 T and transformed in *Escherichia coli*. GST-fused proteins were obtained from total bacterial lysates. Glutathione Agarose 4B beads (Protino®) were coated with GST-fused CRAF-RBD and PI3K-RBD and GTP-bound RAS proteins were pulled down from total cell lysates and were probed by western blot. RAS paralogs were detected by validated antibodies: mouse anti-pan-RAS (Millipore, #05–516), mouse anti-KRAS (Sigma, WH0003845M1), mouse anti-NRAS (Santa Cruz, sc-31) and rabbit anti-HRAS (Santa Cruz, sc-520).

### Statistical analysis

All assays were carried out in three independent experiments in duplicates and triplicates. Data were analyzed by one-way analysis of variance (ANOVA). Differences in treatment levels were further evaluated for significance with Tukey post hoc comparisons. A level of *P* < 0.05 was considered significant. Statistical analysis was performed using SPSS software (SPSS v.20). Values are expressed as mean ± SD.

## Results

### Undifferentiated state of hiPSCs

**Fig. 1:**
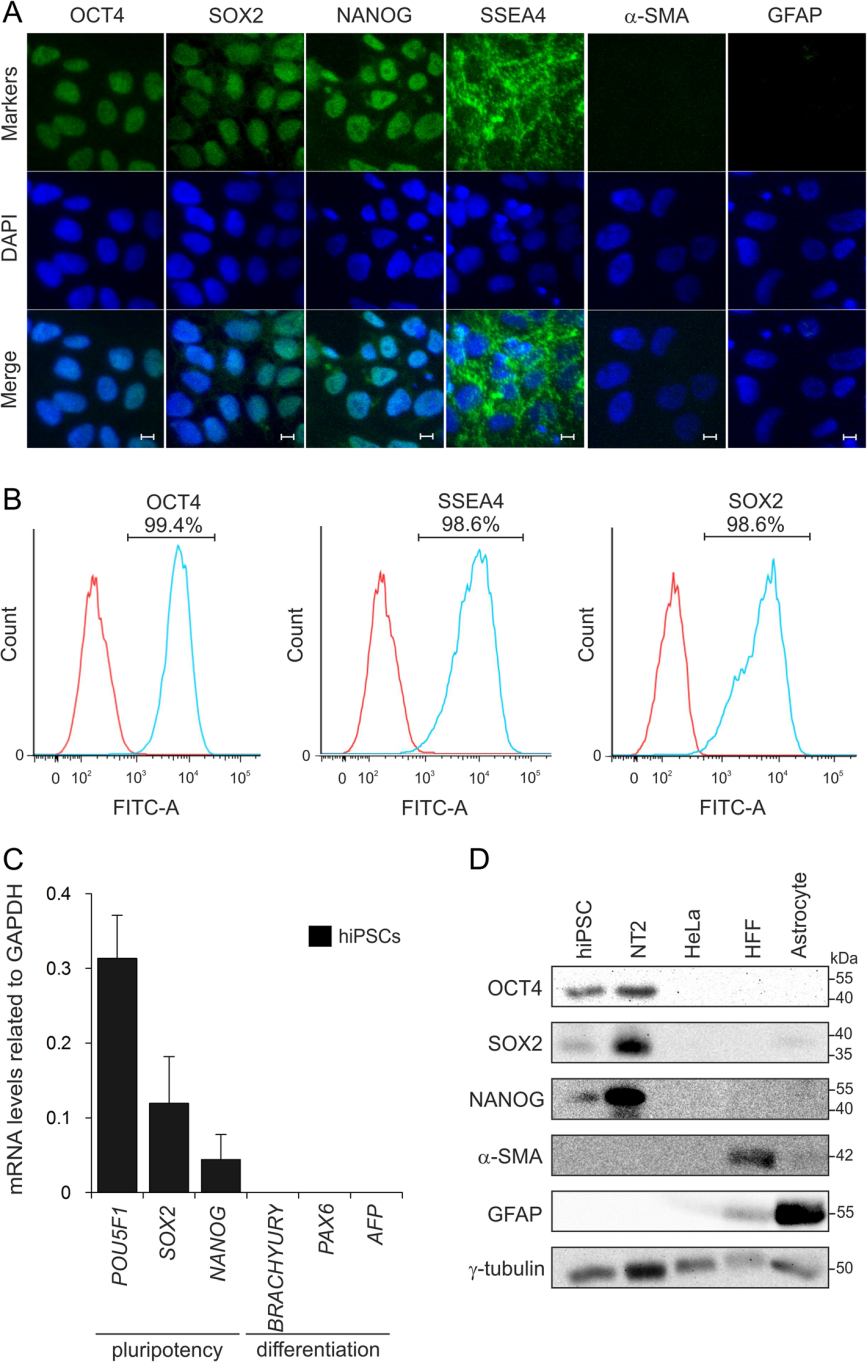
Long-term maintenance of undifferentiated hiPSCs. **a** Confocal imaging showed the expression of pluripotency markers (OCT4, SOX2, NANOG and SSEA4) and the absence of differentiation markers GFAP and α-SMA as ectodermal and mesodermal markers, respectively. Cell nuclei were stained with DAPI (blue). Scale bars, 10 μm. **b** Flow cytometry confirmed expression of OCT4, SSEA4 and SOX2 in hiPSCs with more than 98% of positive cells. **c** qPCR analysis for undifferentiated stem cell markers (*POU5F1*, *SOX2* and *NANOG*) and early commitment to differentiation markers (*BRACHYURY*, *PAX6* and *AFP*). *GAPDH* was used as an internal control. **d** Immunoblot analysis showing the specificity of antibodies and expression of markers. HeLa cells were used as negative control, HFF and astrocytes were used as positive control for α-SMA and GFAP, respectively

To ensure stable conditions, hiPSCs were expanded on Geltrex-coated dishes with conditioned medium (CM) from iMEF supplemented with 100 ng/ml bFGF (see methods). We first analyzed the expression of pluripotency and differentiation markers at the mRNA and protein levels. Confocal imaging of hiPSCs revealed the presence of OCT4, SOX2, NANOG and SSEA4 and the absence of differentiation markers α-SMA (mesoderm) and GFAP (ectoderm) [26] in hiPSCs (Fig. 1a). Accordingly, flow cytometry data showed that more than 98% of the cells were positive for stemness makers, including OCT4, SOX2 and SSEA4 (Fig. 1b). Early and spontaneous lineage specific markers of mesoderm (*BRACHYURY*)*,* ectoderm (*PAX6*) and endoderm (*AFP*) [26] were greatly absent at the mRNA level, while pluripotency genes were expressed (Fig. 1c). Western blot analysis verified the presence of OCT4, SOX2 and NANOG in hiPSCs and NT2 cells (pluripotent embryonal carcinoma cells, as a positive control), as well as the absence of α-SMA and GFAP (Fig. 1d). HeLa cells were used as negative control for all proteins and HFF cells and astrocytes were used as positive controls for α-SMA and GFAP, respectively (Fig. 1d). These data clearly confirmed that hiPSCs were undifferentiated for at least 15 passages under culture conditions using CM-100.

### bFGF maintains undifferentiated state of hiPSCs

**Fig. 2:**
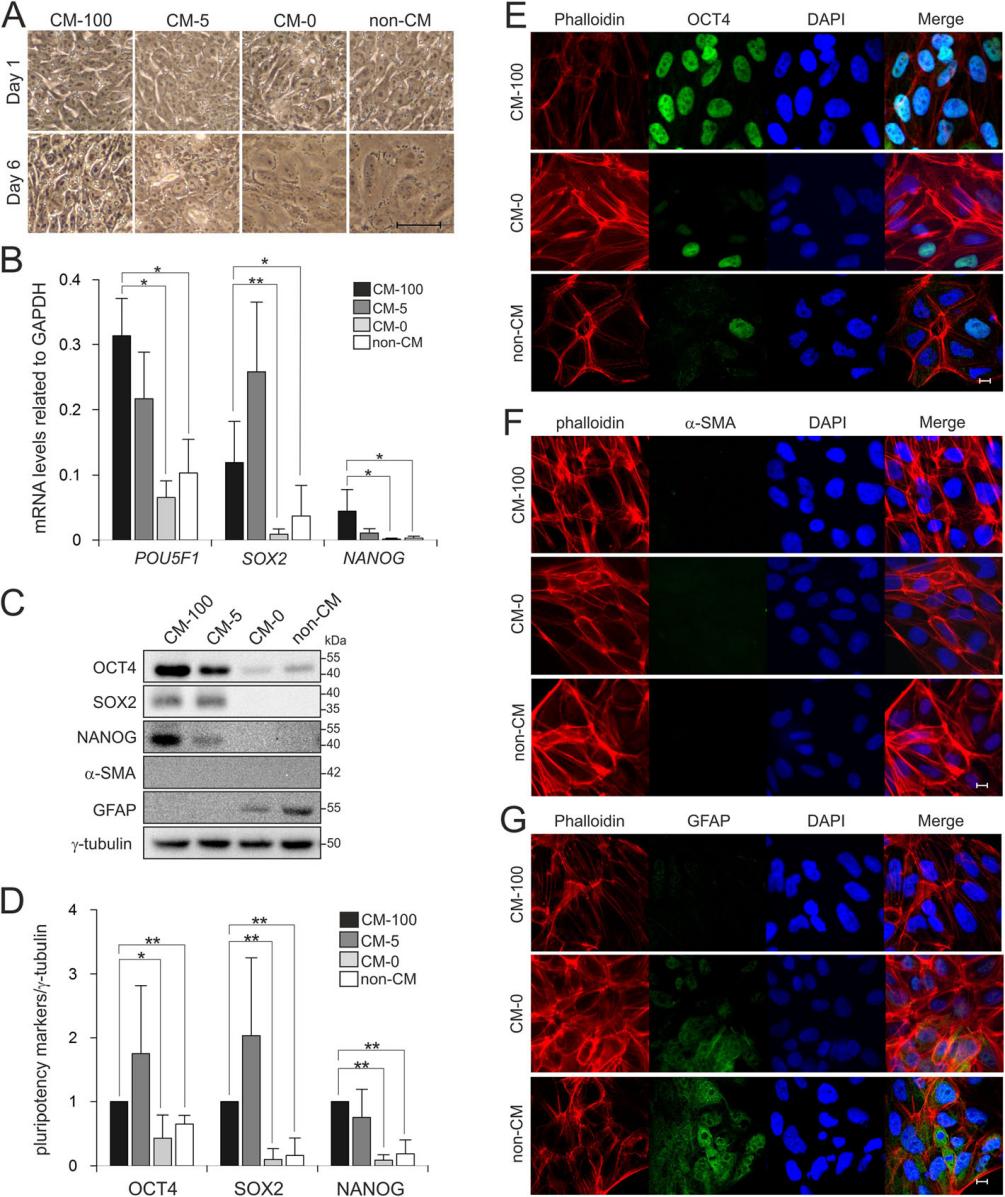
The critical role of bFGF for maintaining hiPSC pluripotency. **a** Phase contrast images of hiPSCs cultured under four different conditions, CM-100, CM-5, CM-0 and non-CM for 6 days. Undifferentiated hiPSCs formed compact colonies (CM-100), while without bFGF supplementation hiPSCs spread and flattened at day 6 (CM-0 and non-CM). Scale bar, 50 μm. **b** qPCR analysis showed the downregulation of pluripotency markers *POU5F1*, *SOX2* and *NANOG* in cells cultured in CM-0 and non-CM in comparison to control group (CM-100). All expression values were normalized to *GAPDH*. Results from three separate experiments, each carried out in triplicate, are shown as mean ± SD (ANOVA; *p < 0.05 and **p < 0.01). **c** Western blot analysis of pluripotency and differentation markers under four different conditions showed the downregulation of stemness markers and upregulation of GFAP. γ-tubulin served as loading control. **d** The graph represents densitometric analysis of three independent experiments, each carried out in duplicates. All values were normalized to γ-tubulin and relative to CM-100. Data are shown as mean ± SD (ANOVA; *p < 0.05 and **p < 0.01). hiPSCs were immunostained for OCT4 (**e**), α-SMA (**f**) and GFAP (**g**) under three different culture conditions; CM-100, CM-0 and non-CM. Cell nuclei were stained with DAPI (blue) and F-actin was stained with phalloidin (red). Scale bar, 10 μm

In order to investigate the effect of bFGF on hiPSCs, feeder-free culture conditions were used to eliminate indirect effects of fibroblasts on hiPSCs. hiPSCs were cultured for 6 days under four different medium conditions containing various bFGF concentrations, i.e. CM-100, CM-5, CM-0 and non-CM used as a negative control. As indicated in Fig. 2a and Additional file 1: Figure S1, withdrawal of bFGF disrupted the compact morphology of hiPSCs, which spreaded out at day 6. These morphological changes are often correlated with the loss of pluripotency [1]. Therefore, we assessed the pluripotent state of the cells by determining OCT4, SOX2 and NANOG expression at mRNA and protein levels. qPCR data revealed a significant reduction in *POU5F1*, *SOX2* and *NANOG* expression 6 days after withdrawing bFGF (CM-0 and non-CM) as compared to CM-100 (Fig. 2b). Consistent with the mRNA expression data, the amount of OCT4, SOX2 and NANOG proteins were drastically and significantly reduced in the absence of bFGF (Figs. 2c, d). Interestingly, loss of pluripotency markers under CM-0 and non-CM at day 6 (Figs. 2b, c) were followed by the expression of GFAP, but not α-SMA, as differentiation marker (Fig. 2c). Moreover, confocal imaging also confirmed the reduction in OCT4 expression and differentiation toward the ectoderm and not mesoderm lineage in hiPSCs (Figs. 2e-g). Thus, bFGF is essential for maintaining pluripotency in hiPSCs and removing it from the culture medium leads to cell differentiation towards the ectoderm lineage.

### MAPK pathway is required for maintaining hiPSCs in an undifferentiated state

**Fig. 3:**
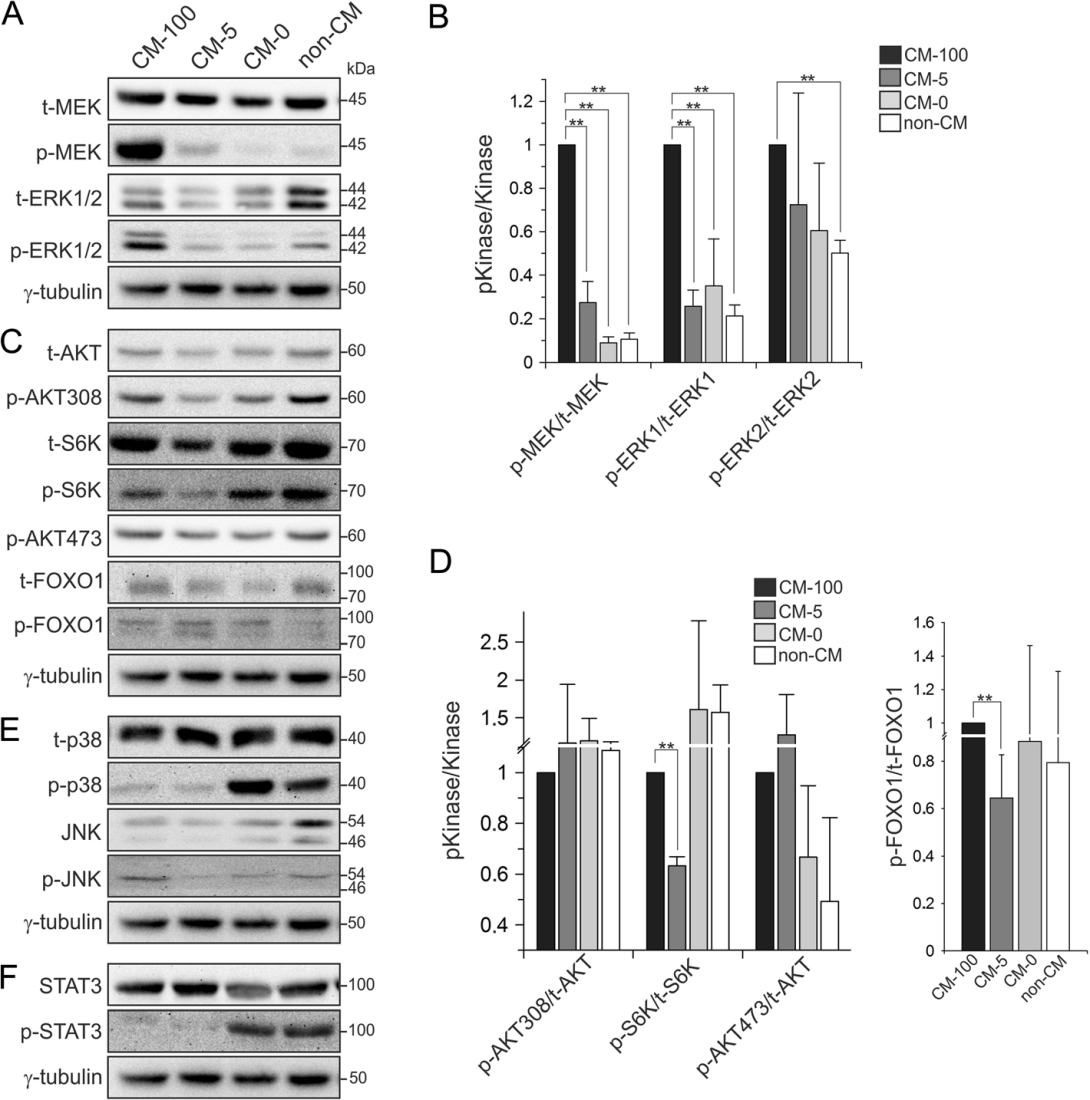
Signaling pathways downstream of bFGF in undifferentiated and differentiated hiPSCs. **a** Immunoblot analysis of the component of the MAPK pathway, including p-MEK and p-ERK1/2 in hiPSCs in undifferentiated (CM-100) and differentiated hiPSCs (CM-0 and non-CM). Total amounts of MEK and ERK1/2 as well as γ-tubulin served as loading controls. **b** The graph represents densitometric analysis of three independent experiments, each carried out in duplicates. All values were normalized to γ-tubulin and relative to CM-100. Data are shown as mean ± SD (ANOVA; **p < 0.01). **c** Immunoblot of the phosphorylated signaling proteins downstream of the PI3K-AKT and mTORC2 axis in undifferentiated (CM-100) and differentiated hiPSCs (CM-0 and non-CM). Total amounts of AKT, S6K, FOXO1 as well as γ-tubulin served as loading controls. **d** The graph represents densitometric analysis of three independent experiments, each carried out in duplicates. All values were normalized to γ-tubulin and relative to CM-100. Data are shown as mean ± SD (ANOVA; **p < 0.01). **e** Immunoblot of other MAPK pathways indulding p-p38 and p-JNK. Total amounts of p38, JNK and γ-tubulin served as loading controls. **f** Immunoblot analysis of p-STAT3 in undifferentiated (CM-100) and differentiated hiPSCs (CM-0 and non-CM). Total amounts of STAT3 and γ-tubulin served as loading controls

Conflicting studies have shown that MAPK pathway can positively and negatively regulate hESCs pluripotency [7, 27]. Thus, we first analyzed the activation status of this pathway. As shown in Fig. 3a (first lane from left), the MAPK pathway was highly active in undifferentiated hiPSCs (CM-100), as detected by immunoblotting of phosphorylated (p-) MEK and ERK1/2 proteins. This pathway is activated by bFGF [7] and given that bFGF is essential for maintaining undifferentiated state of hiPSCs (Figs. 2b, c), we next analyzed the MAPK pathway activity in pluripotent vs. differentiated hiPSCs. Our data showed that following withdrawal of bFGF (CM-0 and non-CM as compared to CM-100) the activity of MAPK pathway was reduced by 10- and 2-fold, as seen by decreased levels of p-MEK and p-ERK1/2, respectively (Figs. 3a, b). Thus, these data indicate that MAPK pathway activity downstream of bFGF is required to keep hiPSCs in a pluripotent state.

### PI3K/AKT pathway remains unchanged during hiPSCs differentiation

Another main signaling pathway downstream of bFGF is PI3K/AKT [18], which promotes activin A and Smad2/3-mediated self-renewal of hPSCs [27] and is essential for hiPSCs survival [28]. Therefore, we examined the signaling activity of bFGF in undifferentiated and differentiated hiPSCs towards AKT and their downstream components S6K and FOXO1. Our data showed that AKT was activated in undifferentiated hiPSCs (CM-100) via both PI3K-PDK1-AKT and mTORC2-AKT pathways as monitored by p-AKT^T308^ and p-AKT^S473^ levels, respectively (Fig. 3c). The downstream components of these respective pathways were also phosphorylated (p-S6K^T389^ and p-FOXO1^S256^; Fig. 3c, first lane from left), presumably resulting in S6K activation and FOXO1 inhibition. However, we did not detect significant changes in the activity of the PI3K-PDK1 and mTORC2 pathways during bFGF withdrawal induced differentiation of hiPSC cells (Fig. 3c, d). Thus, PI3K-AKT signaling pathways are most probably involved in the control of cell survival rather than the maintenance of hiPSCs pluripotency.

### p38 and STAT3 activation during hiPSCs differentiation

In the next step, we monitored the activation state of p38, JNK and STAT3, respectively, as further candidate pathways downstream of FGF [18]. We found that p38 and STAT3 were not fully active in undifferentiated hiPSCs (CM-100) (Figs. 3e, f). Interestingly, however, following withdrawal of bFGF, the levels of phosphorylated and activated p-Thr180/Tyr182 p38 and p-Tyr705 STAT3, were clearly increased (Figs. 3e, f). Analysis of the JNK pathway showed no changes during differentiation (Fig. 3e). Taken together, p38 and STAT3, but not JNK, are activated upon bFGF withdrawal and may play a role in hiPSCs differentiation.

### Dominant cytoplasmic localization of p-ERK

**Fig. 4:**
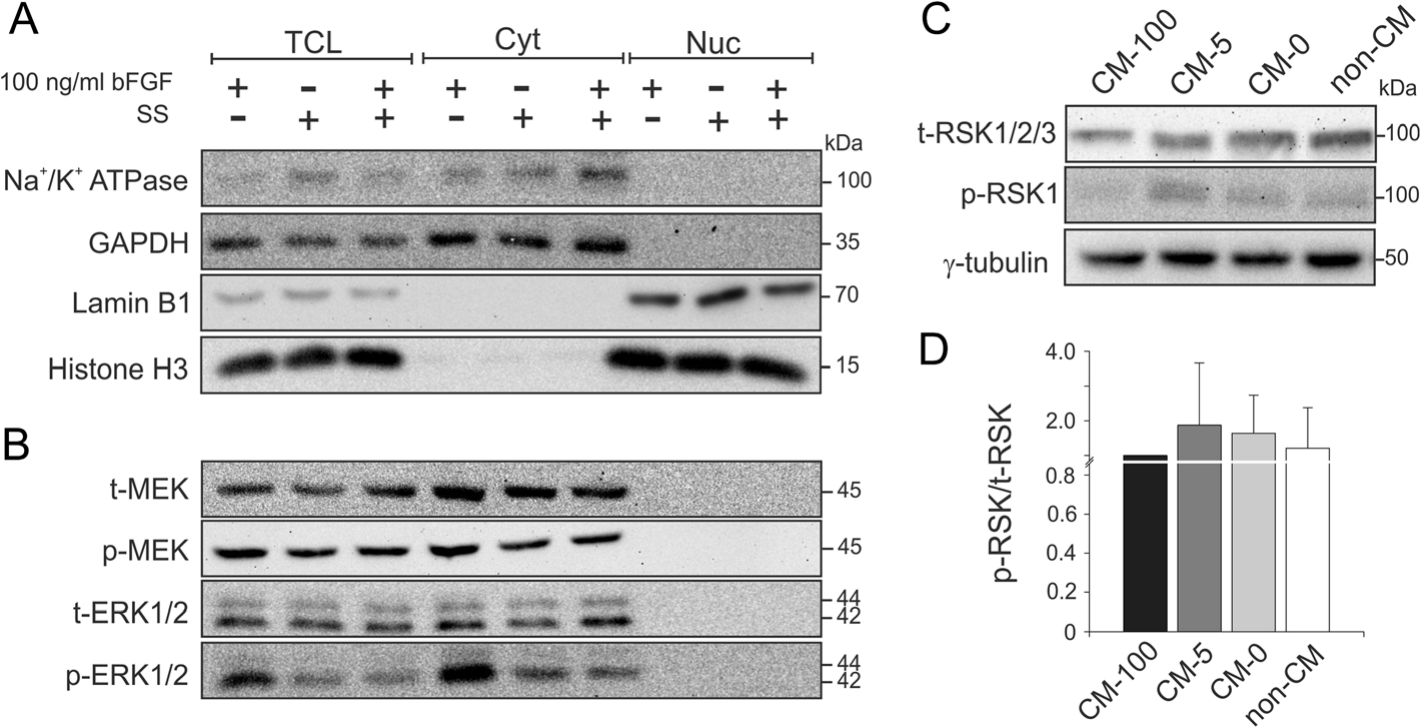
Cellular localization of components of MAPK pathway in undifferentiated hiPSCs. **a** Simple nucleocytoplasmic fractionation. hiPSCs were fractionated into three distinct fractions (TCL, total cell lysate; Cyt, cytosolic fraction; Nuc, nuclear fraction) by using rapid non-ionic detergent-based purification technique. **b** Immunoblot analysis of components of MAPK pathway showed the dominant localization of all proteins in the cytosolic fraction in undifferentiated hiPSCs, before and after bFGF stimulation. **c** Immunoblot analysis of p-RSK in undifferentiated (CM-100) and differentiated hiPSCs (CM-0 and non-CM). Total amounts of RSK and γ-tubulin served as loading controls. **d** The graph represents densitometric analysis of three independent experiments, each carried out in duplicates. All values were normalized to γ-tubulin and relative to CM-100. Data are shown as mean ± SD

Our data indicate that the MAPK signaling is a prominent pathway downstream of bFGF, which maintains hiPSCs pluripotency and the activity will be decreased upon hiPSCs differentiation. Activation and nuclear translocation of MAPKs is necessary to initiate transcriptional programmes controlling cellular responses [29]. Thus, first we analyzed the nucleocytoplasmic distribution of these kinases in undifferentiated hiPSCs by subcellular fractionation. Total cell lysates (TCL) as well as cytosolic (Cyt) and nuclear (Nuc) fractions were analyzed for purity using antibodies directed against specific marker proteins (Fig. 4a). GAPDH and Na^+^/K^+^ ATPase were consistently found in the cytosol and histone H3 and lamin B1 in the nuclear fraction, confirming the purity of each fraction. Remarkably, both p-MEK/MEK and p-ERK1/2/ERK1/2 showed a predominantly cytosolic localization in undifferentiated hiPSCs (Fig. 4b, first lane from left). Subcellular fractionation was performed using another clone of hiPSCs and NT2 cells and comparable results were obtained (Additional file 1: Figure S3). Moreover, cells were starved for 12 h and then stimulated with 100 ng/ml bFGF for 15 min to observe any changes in p-ERK1/2 distribution upon stimulation. Interestingly both p-MEK/MEK and p-ERK1/2/ERK1/2 showed cytosolic localization even after bFGF stimulation, rather than nuclear localization (Fig. 4b). Consistent with this unexpected result, we investigated the phosphorylation of RSK, a cytosolic target of p-ERK1/2 that previously has been reported in hESCs [30, 31]. Our data revealed phosphorylation of RSK (at position T573) during hiPSCs differentiation (CM-0 or non-CM as compared to CM-100) (Figs. 4c, d), which is not consistent with predominant cytosolic localization of p-ERK1/2 (Fig. 4b).

### NRAS as an upstream regulator of undifferentiated hiPSCs

**Fig. 5:**
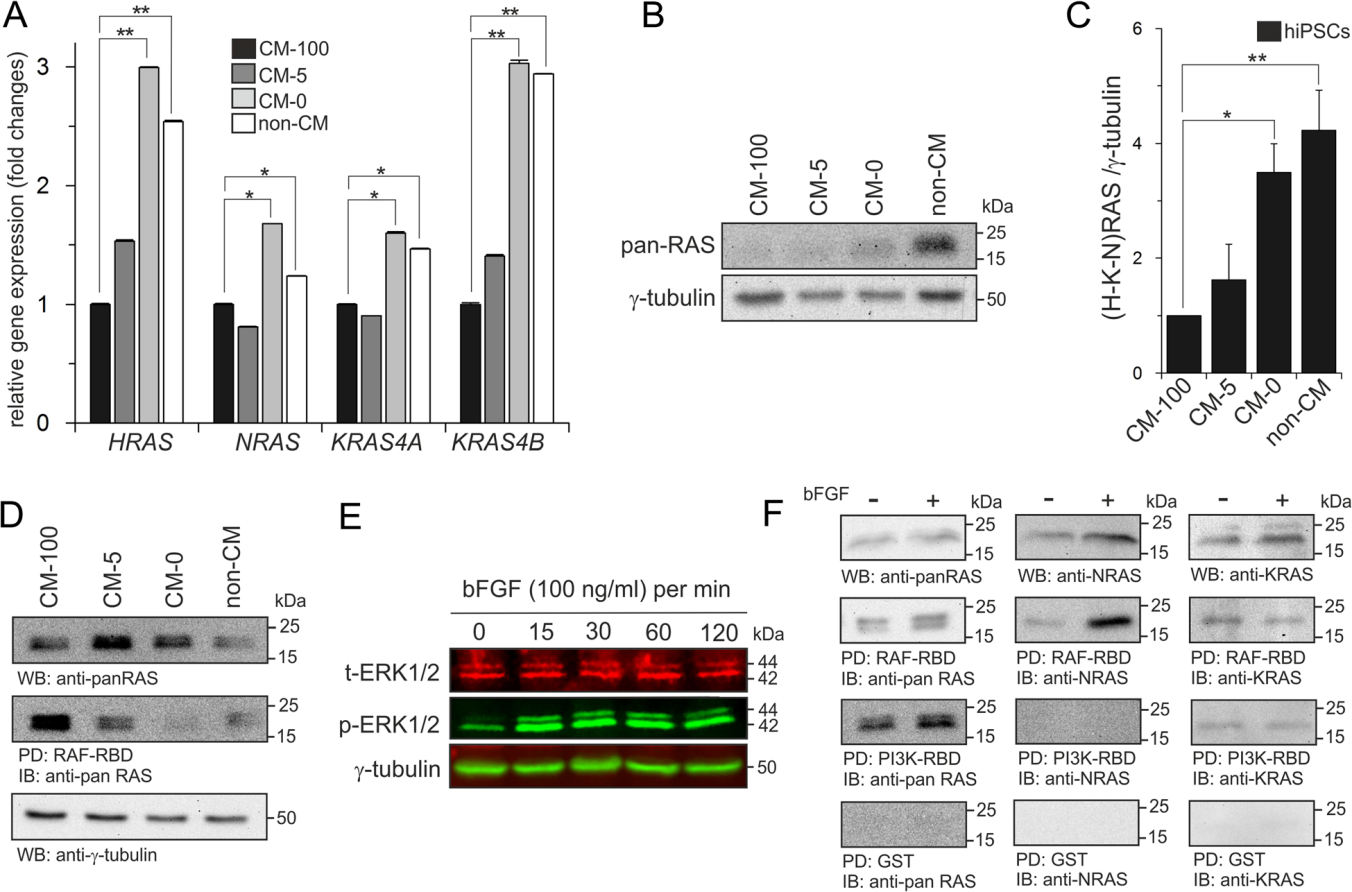
RAS paralogs display different expression and activation patterns in undifferentiated vs. differentiated hiPSCs. **a** qPCR analysis of RAS-related genes in undifferentiated vs. differentiated hiPSCs. Results from three separate experiments, each carried out in triplicate, are shown as mean ± SD (ANOVA; *p < 0.05 and **p < 0.01). **b** Immunoblot analysis of RAS protein expression in undifferentiated (CM-100) and differentiated hiPSCs (CM-0 and non-CM). **c** The graph represents densitometric analysis of three independent experiments, each carried out in duplicates. All values were normalized to γ-tubulin and relative to CM-100. Data are shown as mean ± SD (ANOVA; **p < 0.01). **d** CRAF-RBD-derived pull-down of GTP-bound RAS in differentiated (CM-0 and non-CM) and undifferentiated (CM-100) hiPSCs were analyzed by immunoblotting of pan-RAS. γ-tubulin was used as loding control. **e** Immunoblot analysis of p-ERK1/2 showed that transient bFGF stimulation induces phosphorylation of ERK1/2 in KSR-starved hiPSCs after 15 min. The amounts of ERK1/2 and γ-tubulin were used as loading controls. **f** RAS paralogs were subjected to pull-down analysis in undifferentiated hiPSCs in the presence and absence of bFGF using two GST-fused RAS effectors, CRAF-RBD and PI3Kα-RBD. RAS-GTP pull down assays were followed by western blot analysis using pan-RAS antibody and RAS paralog specific antibodies. Immunoblots of total cell lysates were served as loading controls to detect pan-RAS, NRAS and KRAS. GST and pan-RAS were used as negative and loading controls, respectivley

Small GTP-binding proteins of the RAS family transduce extracellular signals and activate a multitude of pathways via activation of effector proteins [32]. RAF kinases and PI3Ks are well-studied effectors of RAS family members which in turn activate MAPK and AKT, respectively [32]. As our data indicate that MAPK pathway is involved in maintaining pluripotency and PI3K/AKT likely involved in the survival of hiPSCs, we further investigated the RAS paralogs specificity downstream of bFGF and upstream of these pathways. First, the expression profile of three RAS paralogs (H/N/K) was investigated at both mRNA and protein levels in undifferentiated vs. differentiated hiPSCs. All four RAS genes were expressed and upregulated (3-fold for *HRAS* and *KRAS4B* and approximately 2-fold for *NRAS* and *KRAS4A*) upon differentiation (CM-0 and non-CM) (Fig. 5a). Interestingly, the amount of RAS proteins in differentiated hiPSCs (CM-0 and non-CM) was also increased 4-fold as compared to undifferentiated cells (CM-100) (Fig. 5b, c). To gain insights into the level of RAS activity (GTP-bound), total cell lysates of undifferentiated (CM-100) and differentiated (CM-0 and non-CM) hiPSCs were prepared and pull-down assays were performed with CRAF-RBD as RAS effector protein. As indicated in Fig. 5d, RAS activity was drastically reduced upon differentiation (CM-0 and non-CM) in comparison to the undifferentiated state (CM-100) that was in consistent with the reduction in MAPK pathway activity (Fig. 3a).

To examine the levels of active (GTP-bound) RAS paralogs, undifferentiated hiPSCs were serum starved for 12 h and re-stimulated with 100 ng/ml bFGF for different time points. Based on the phosphorylation levels of p-ERK1/2 (Fig. 5e), stimulation with bFGF for 15 min was chosen. For pull-down analysis, two major RAS effector proteins were employed; CRAF-RBD and PI3K-RBD which were used as GST-fusion proteins. As indicated in Fig. 5f, RAS activity was increased upon bFGF stimulation leading to a stronger RAF activation as compared to PI3K. To further investigate the activity of each of the three canonical RAS proteins, we used paralog-specific antibodies (Additional file1: Figure S4) [33]. Interestingly, we found that NRAS is the main RAS paralog which is activated upon bFGF stimulation (Fig. 5f). It preferentially and most strongly bound to CRAF as compared to PI3K for which no detectable binding was observed (Fig. 5f). KRAS bound to CRAF showed no elevated activity upon bFGF stimulation (Fig. 5f). The expression level of HRAS protein was very low in hiPSCs and was not detectable (data are not shown). Altogether, these data suggest that NRAS acts as a main RAS paralog that links bFGF signaling to the MAPK pathway.

## Discussion

**Fig. 6:**
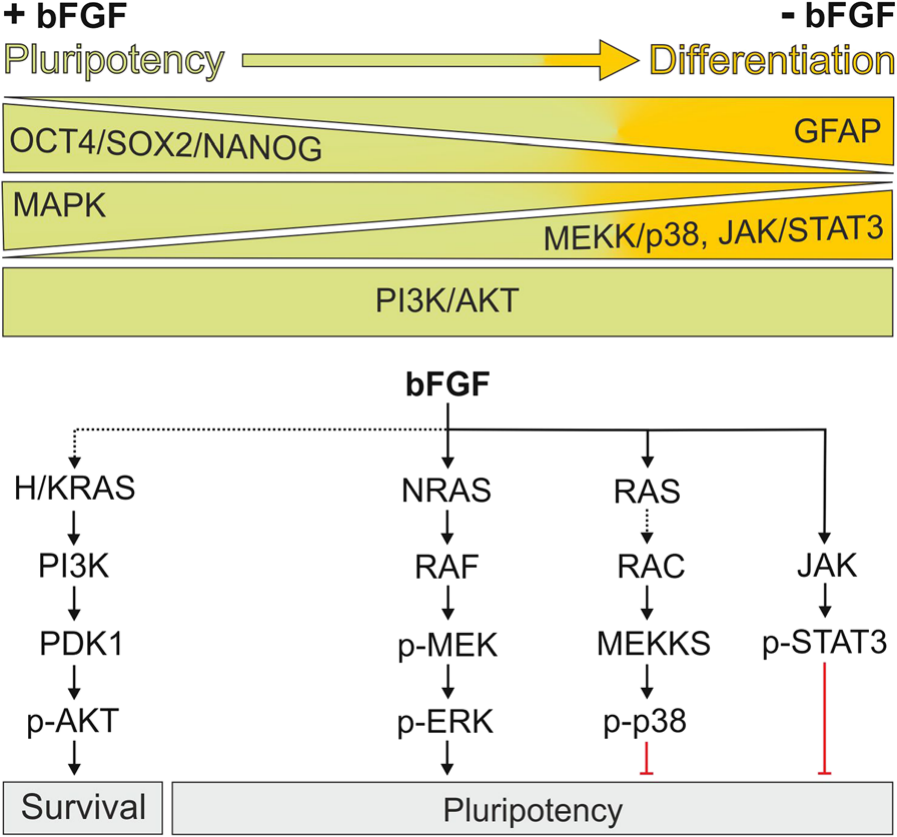
Proposed model for bFGF signaling in pluripotent (green) vs. differentiated (orange) hiPSCs (for details see discussion). bFGF, basic fibroblast growth factor; OCT4, octamer-binding transcription factor 4; SOX2, sex determining region Y-box 2; GFAP, glial fibrillary acidic protein; RAS, rat sarcoma; PI3K, phosphoinositide 3-kinase; PDK1, 3-phosphoinositidedependent protein kinase; MEK, MAP/ERK kinase; ERK, extracellular signal-regulated kinase; JAK, Janus kinase; STAT3, signal transducer and activator of transcription 3

This study provides novel molecular insight into the regulation of pluripotency maintenance of hiPSCs. Our findings indicate that among the signaling pathways downstream of bFGF, the MAPK pathway plays a critical role in maintaining pluripotency, whereas strong activation of p38 and JAK/STAT3 signaling is linked to differentiation of hiPSCs. In contrast, no relevant changes occurred in the activation of AKT or JNK pathways from pluripotent hiPSCs towards differentiated cells. Moreover, we identified NRAS among the RAS paralogs as the likely link between bFGF receptor and the MAPK pathway that maintains hiPSCs pluripontency (Fig. 6).

Our data clearly suggest that bFGF transmits signals to promote pluripotency and suppress differentiation activities in hiPSCs (Fig. 6). We showed that bFGF withdrawal from the culture, markedly decreased OCT4, SOX2 and NANOG expression at the mRNA and protein levels, which was followed by the expression of GFAP and differentiation towards the ectoderm lineage. Consistent with our finding in hiPSCs, it was reported that bFGF also maintains hESCs in an undifferentiated state [11, 34].

Different studies suggest pleiotropic effects of bFGF activating different pathways in hESCs either directly or indirectly by inducing paracrine signaling via iMEFs in coculture [35]. For investigating the mechanistic effects of bFGF, we compared the signaling pathways in undifferentiated vs. differentiated hiPSCs obtained via bFGF withdrawal. FGF has been reported to activate multiple downstream signaling pathways, including MAPKs (ERK, JNK and p38), PI3K and JAK/STAT [36]. Our study demonstrates the activation of MEK-ERK1/2 pathway in undifferentiated hiPSCs and a remarkable decrease in the p-MEK and p-ERK1/2 levels by withdrawing bFGF which induces their differentiation. Previously, Li and colleagues have shown that inhibiting FGF signaling induces hESC differentiation into primitive endoderm and trophectoderm [7]. However, Singh et al. have reported a pro-differentiation role of MAPK pathway in hESCs [27]. These conflicting reports could be due to different culture conditions, cell lines or even pathway dose-dependency. Our data argue against a pro-differentiation role of the MAPK pathway. We used in this study a system for culturing hiPSCs with iMEF-CM that was supplemented with 100 ng/ml bFGF which was different from Li et al. and Singh et al. [7, 27]. Under these conditions we are able to dissect direct and paracrine iMEF-mediated influences of bFGF without the risk of confounding effects based on sample contamination with feeder cells. Our data clearly showed that MAPK pathway positively regulates hiPSC pluripotency. Culturing cells with CM-0 and non-CM, which led to differentiation toward ectoderm, was in correlation with a significant decrease in MAPK pathway activity between all groups (CM-100, CM-5, CM-0 and non-CM). As previously described, bFGF activates ERK1/2 at high concentrations (about 100 ng/ml) in ESCs [27]. Here, we observed no obvious differences between high dose (CM-100) and low dose (CM-5) of bFGF in maintaining the undifferentiated status of hiPSCs. Thus, the downregulation of ERK precedes the decrease of pluripotency markers and cannot be excluded under long-term culture (6 days) conditions. These observations suggest that homeostasis of MAPK signaling is a dose-dependent consequence of bFGF without direct impact on the expression level of pluripotency-associated transcription factors.

PI3K/AKT activation by bFGF has also been shown to be important for the maintenance of the undifferentiated state of hESCs [37]. This pathway contributes to a variety of important cellular processes including nutrient uptake, anabolic reactions, proliferation and survival [38]. Proliferation and survival can be controlled by mTORC1 mediated activation of S6K and mTORC2 mediated inhibition of FOXO-1, respectively [39, 40]. Armstrong and colleagues have shown that PI3K/AKT is important for maintaining pluripotency in hES-NCL1 cells and the key components of this pathway, such as p-PDK1, p-PTEN, p-AKT^308^ and p-AKT^473^ are downregulated during differentiation to embryoid bodies [30]. Li and coworkers have shown that PI3K/AKT pathway, downstream of bFGF, is highly active in hESCs, such as H1 and H9 cells, which supports hESC self-renewal and pluripotency [7]. Other studies have implicated the survival and anti-apoptotic role of PI3K/AKT in hESCs and hiPSCs [27, 28, 41]. In our study, two axes of AKT activation were investigated, PI3K-PDK1-AKT-S6K and mTORC2-AKT-FOXO1 as downstream pathways of bFGF, which is different from previous reports that just showed the importance of PI3K/AKT in maintenance of pluripotency and not as a target of bFGF signaling [27, 28, 41]. Our results showed that there was no change in the activation level of these two pathways following hiPSCs differentiation (CM-0 and non-CM). This suggests that AKT-S6K and AKT-FOXO1 signaling remain unaffected in the presence and absence of bFGF during a long-term culture (6 days) which may be due to the presence of KSR in iMEF-CM. KSR contains high levels of insulin that can activate AKT pathways [27]. This rather suggests that PI3K/AKT is not critical for maintaining the undifferentiated state of hiPSCs and most probably plays an anti-apoptotic role required for survival of hiPSCs rather than their pluripotency.

In addition to MAPK and PI3K/AKT pathways, we also analyzed other signaling pathways, including p38 MAPK and JNK (c-Jun N-terminal kinase), both can be activated by FGF signaling [35]. p38 activation has been observed in response to a variety of extracellular stresses and mitogenic stimuli which lead to different cell-specific responses, including inflammation, cell death, senescence, survival, cell growth and differentiation [42]. So far, little is known about the role of p38 in pluripotency of hESCs. Neganova and colleagues demonstrated an increased activity of p38 MAPK during the early stage of reprogramming of human fibroblasts to hiPSCs and the importance of this pathway for obtaining fully reprogrammed cells [43]. Moreover, hESCs and hiPSCs are in a high-methionine metabolic state which decreases upon differentiation. In this regard, it has been shown that methionine deprivation triggering the activation of p53-p38 signaling leads to NANOG downregulation and differentiation into all three germ layers [44]. We showed in this study, for the first time, an increase in p38 MAPK activity during hiPSCs differentiation under bFGF starvation (CM-0). It can be proposed that p38 is inhibited as a downstream target of bFGF in undifferentiated hiPSCs.

Findings from *Drosophila* studies and some human cancers indicate that JNK might be a regulator of stem cells and cancer stem cells. Brill et al. observed a significantly elevated JNK activity in undifferentiated hESCs, which if blocked by JNK inhibitors under feeder-free conditions in the presence of CM, leaded to decreased OCT4 expression and differentiation [45]. A possible contribution of JNK signaling to the maintenance and/or self-renewal of hESCs was additionally confirmed in a different hESC line, Harvard’s HUES-7. In response to BMP-induced differentiation, a transient elevation of c-Jun phosphorylation was observed, which indicates both the competence of the basal JNK pathway to maintain the stemness of the hESCs and a possible involvement of JNK activation in the initiation of hESC differentiation [46]. In our study, we observed the constant activation of JNK during hiPSCs differentiation in response to bFGF starvation. Thus, JNK pathway may be involved in other cellular responses rather than maintaining pluripotency or inducing differentiation.

mESCs can be maintained in vitro by adding leukemia inhibitory factor (LIF) to the medium and its withdrawal rapidly leads to differentiation [47, 48]. LIF activates Janus kinases (JAKs) which subsequently phosphorylate STAT3. Activated STAT3 translocates into the nucleus and activates transcription of target genes [49, 50]. Interestingly, LIF/STAT3 signaling fails to support self-renewal of hESCs and is nonresponsive to LIF/STAT3 [51]. Since LIF is not the only cytokine that activates JAK/STAT3 pathway, we analyzed the activity of this pathway downstream of bFGF. Similar to Humphrey and coworkers, who have shown that STAT3 phosphorylation was not detectable in undifferentiated hESCs [52], we also could not observe phosphorylation of STAT3 in undifferentiated hiPSCs. Interestingly, upon differentiation (CM-0 and non-CM), JAK/STAT3 pathway was activated in hiPSCs. It can be postulated that unlike mESCs, hiPSCs do not require STAT3 activity for the maintenance of their pluripotency but rather for their differentiation.

It has been shown that activated ERKs in hESC are translocated from the cytoplasm to the nucleus where they phosphorylate and activate nuclear transcription factors and effectors, such as ELK1 and MYC [30]. These downstream targets of MAPK pathway are downregulated during differentiation [30]. We performed subcellular fractionation of undifferentiated hiPSCs to analyze the cellular distribution of MAPK pathway components. Both p-MEK and p-ERK1/2 were located in the cytoplasm, even after stimulation with bFGF for 15 min. Therefore, we analyzed the activity levels of RSK as one possible cytosolic target of p-ERK1/2 in undifferentiated and differentiated hiPSCs. There was an increase in phosphorylation level of RSK in CM-0 and non-CM compared to CM-100 which was in conflict with the loss of ERK1/2 activity during differentiation of hiPSCs. Thus, these data suggest that p-ERK1/2 in hiPSCs most probably signal through further (critical) cytosolic targets other than RSK.

We demonstrated the critical role of MAPK pathway downstream of bFGF in maintaining pluripotency in hiPSCs. For further analysis of this pathway, we analyzed the expression of canonical RAS isoforms in undifferentiated vs. differentiated hiPSCs. Interestingly we found that in contrast to the decreased level of MAPK pathway activity in differentiated hiPSCs, the levels of RAS mRNA and protein were both upregulated upon differentiation. To elucidate the activity level of RAS (GTP-bound), pull down assays were performed with CRAF-RBD as an effector for RAS proteins. RAS activity was drastically reduced in hiPSCs treated with CM-0 and non-CM (differentiated cells) compared to undifferentiated cells (CM-100), consistent with the decrease of MAPK pathway activity levels. These findings suggest that RAS-RAF is upstream of MEK/ERK and its activity will be decreased upon differentiation in hiPSCs. Furthermore, we analyzed main RAS paralogs, i.e. H-, K- and NRAS. Interaction analyses with two RAS effectors (RAF and PI3K) showed that among the RAS paralogs, NRAS preferentially interacts with RAF in the presence of bFGF and activates the MAPK pathway while no interaction was observed with PI3K independent of the bFGF stimulation status. KRAS interacts physically with RAF and PI3K but showed no preference for either of the effectors upon bFGF starvation or stimulation. Lastly, HRAS was the most difficult RAS to analyze. Although the mRNA levels of HRAS were higher than NRAS in hiPSCs (data not shown), at the protein level, HRAS was not detectable (data not shown).

Signaling pathways regulating ESC fate differ between mESCs and hESCs, and our study provides another aspect of this difference in signal transduction. Recently, Altshuler and colleagues have shown that all three RAS paralogs regulate the transition from naïve to primed state in mESCs and HRAS, KRAS and NRAS display a similar pattern of activation and overlapping roles in mESC differentiation [22]. However, we clearly observed the difference between the patterns of these paralogs in regulating the downstream pathways, which are involved in maintaining pluripotency. Further studies are needed to investigate the role of these RAS proteins in hiPSC differentiation which will provide a better understanding of pluripotency states and early human embryonic development.

## Conclusion

In conclusion, our study suggests that among the downstream pathways of bFGF, MAPK pathway plays a prominent role in keeping hiPSCs in a pluripotent state, while two axes of AKT pathway (PI3K-PDK1-AKT-S6K and mTORC2-AKT-FOXO1) remain unchanged during differentiation that propose a survival role of this pathway rather than maintaining pluripotency which is different from previous reports. Among other pathways, p38 and JAK/STAT3 were activated upon bFGF withdrawal and hiPSCs differentiation, and JNK, like AKT pathway, remain unchanged. Characterizing the MAPK pathway in more detail revealed that among RAS isoforms, NRAS is the link between bFGF receptor and MAPK pathway leads to hiPSCs pluripotency.

## Additional file

Additional file 1: **Table S1.** Primer sequences (5′ to 3′) for qPCR using the SYBR Green system obtained from PrimerBank (https://pga.mgh.harvard.edu/primerbank/). **Figure S1.** Morphological changes of hiPSCs during 6 day culture of bFGF starvation. **Figure S2.** Quantitative western blot analysis of cell signaling in hiPSCs with different culture conditions. **Figure S3.** Subcellular distribution of ERK1/2. **Figure S4.** Specification and validation of RAS antibodies. (DOCX 2260 kb)

## Abbreviations

bFGF: Basic fibroblast growth factor
CM: Conditioned medium
ESC: Embryonic stem cell
GFAP: Glial fibrillary acidic protein
hESC: Human embryonic stem cell
HFF: Human foreskin fibroblast
hiPSC: Human induced pluripotent stem cell
iMEF: irradiated murine embryonic fibroblast
JAK/STAT: Janus kinase/signal transducers and activators of transcription
JNK: c-Jun N-terminal kinases
KSR: Knockout serum replacement
MAPK: Mitogen-activated protein kinase
mESC: Mouse embryonic stem cell
NEAA: Non-essential amino acid
non-CM: Non-conditioned medium
PI3K: Phosphatidylinositol-4,5-bisphosphate 3-kinase
α-SMA: Alpha smooth muscle actin
