# Supplementary material for: bFGF-mediated pluripotency maintenance in human induced pluripotent stem cells is associated with NRAS-MAPK signaling
Source: Cell Commun Signal. 2018 Dec 5;16:96. doi: 10.1186/s12964-018-0307-1 (PMC6282345; doi:10.1186/s12964-018-0307-1)
Supplement: Supplementary file 1 — Table S1. Primer sequences (5′ to 3′) for qPCR using the SYBR Green system obtained from PrimerBank (https://pga.mgh.harvard.edu/primerbank/). Figure S1. Morphological changes of hiPSCs during 6 day culture of bFGF starvation. Figure S2. Quantitative western blot analysis of cell signaling in hiPSCs with different culture conditions. Figure S3. Subcellular distribution of ERK1/2. Figure S4. Specification and validation of RAS antibodies. (DOCX 2260 kb) [file 12964_2018_307_MOESM1_ESM.docx]

**Supplementary Information**

**bFGF-mediated pluripotency maintenance in human induced pluripotent stem cells is associated with NRAS-MAPK signaling**

Fereshteh Haghighi^1^, Julia Dahlmann^2,3^, Saeideh Nakhaei-Rad^1^, Alexander Lang^1#^, Ingo Kutschka^2^, Martin Zenker^4^, George Kensah^2,3^, Roland P. Piekorz^1^, Mohammad Reza Ahmadian^1@^

^1^Institute of Biochemistry and Molecular Biology II, Medical Faculty of the Heinrich Heine University, Düsseldorf, Germany; ^2^Department of Thoracic and Cardiovascular Surgery, University of Göttingen, Germany; ^3^Department of Cardiothoracic Surgery, University Clinic, Otto von Guericke-University, Magdeburg, Germany; ^4^Institute of Human Genetics, Otto von Guericke-University, Magdeburg, Germany.

**Table S1.** Primer sequences (5´ to 3´) for qPCR using the SYBR Green system obtained from PrimerBank (https://pga.mgh.harvard.edu/primerbank/).

| Genes | Forward primers | Reverse primers |
| --- | --- | --- |
| *POU5F1* | CTGGGTTGATCCTCGGACCT | CACAGAACTCATACGGCGGG |
| *SOX2* | GCCGAGTGGAAACTTTTGTCG | GGCAGCGTGTACTTATCCTTCT |
| *NANOG* | CCCCAGCCTTTACTCTTCCTA | CCAGGTTGAATTGTTCCAGGTC |
| *BRACHYURY* | TATGAGCCTCGAATCCACATAGT | CCTCGTTCTGATAAGCAGTCAC |
| *PAX6* | TGGGCAGGTATTACGAGACTG | ACTCCCGCTTATACTGGGCTA |
| *AFP* | CTTTGGGCTGCTCGCTATGA | GCATGTTGATTTAACAAGCTGCT |
| *GAPDH* | GGAAGGTGAAGGTCGGAGTCA | GTCATTGATGGCAACAATATCCACT |
| *HRAS* | CAGATCAAACGGGTGAAGGAC | GCCTGCCGAGATTCCACAG |
| *NRAS* | TGAGAGACCAATACATGAGGACA | CCCTGTAGAGGTTAATATCCGCA |
| *KRAS4A* | ACAGAGAGTGGAGGATGCTTT | TTTCACACAGCCAGGAGTCTT |
| *KRAS4B* | GATTTGCCTTCTAGAACAGTAGACAC | CAACACCCTGTCTTGTCTTTGC |

**
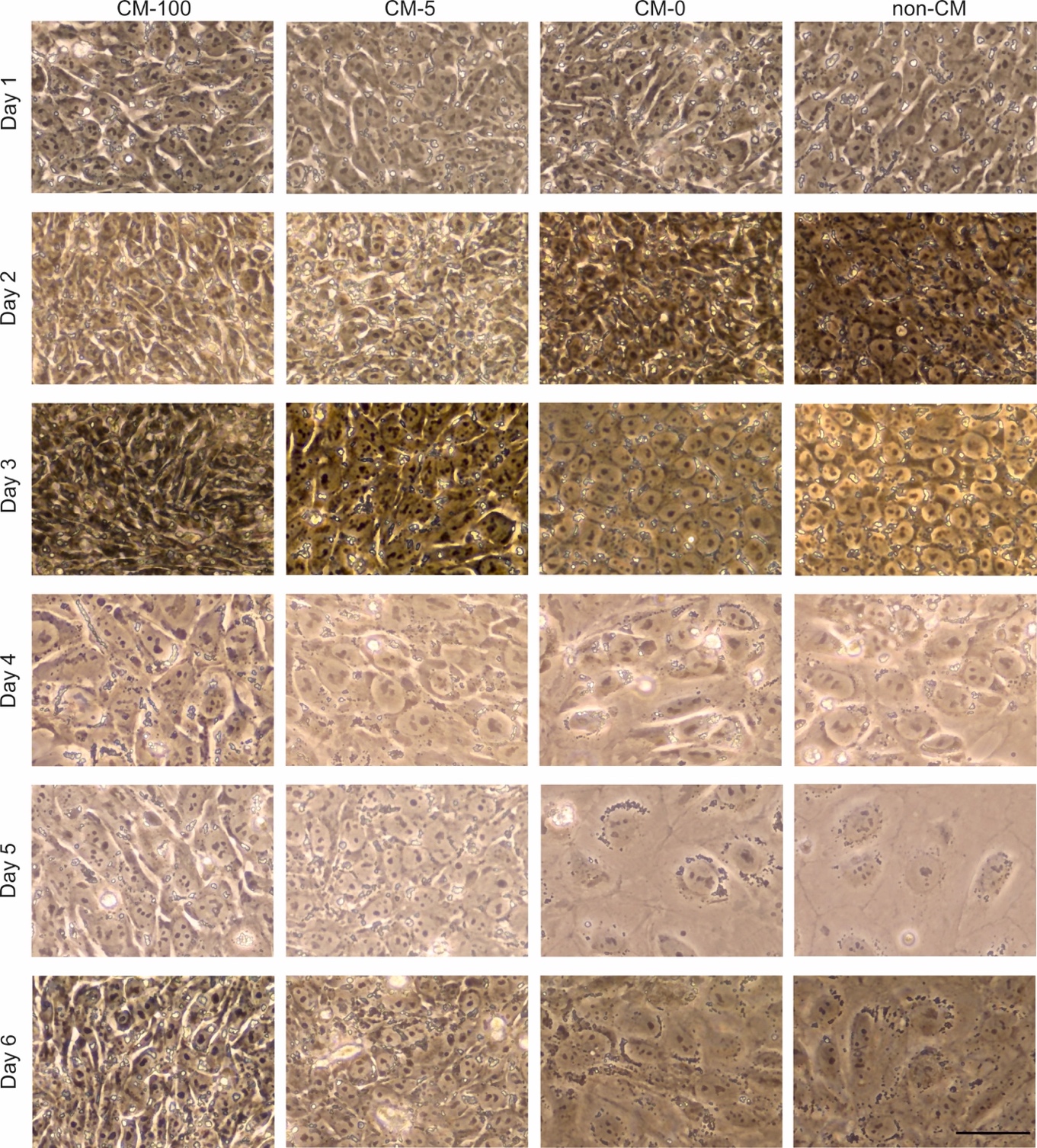
**

**Figure S1: Morphological changes of hiPSCs during 6 day culture of bFGF starvation.** hiPSCs were cultured with CM-100 at the density of 5 x 10^4^ cells/cm^2^. The day after, they were washed with PBS^-/-^ and treated with the CM-100, CM-5, CM-0 and non-CM and were kept in culture for 6 days. Cells treated with CM-0 and non-CM started to spread out and flattened at day 4 which was correlated with the loss of pluripotency. Scale bar, 50 μm.

**
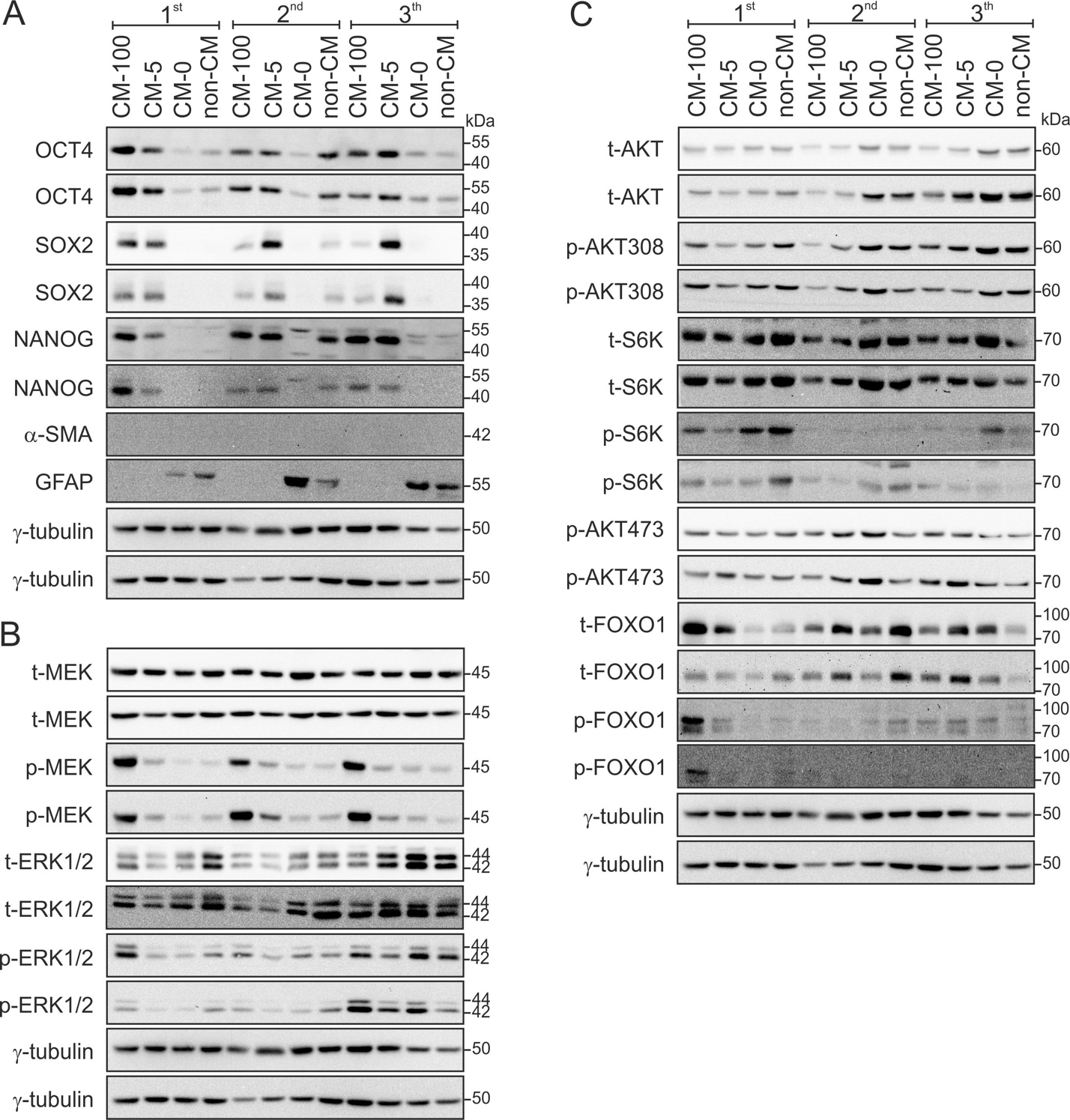
**

**Figure S2: Quantitative western blot analysis of cell signaling in hiPSCs with different culture conditions.** Quantitative western blots were probed with antibodies against pluripotency and differentation markers **(A)**, against components of the RAS-RAF-MEK-ERK pathway (B) and PI3K-AKT-S6K/FOXO1 pathway **(C)**.


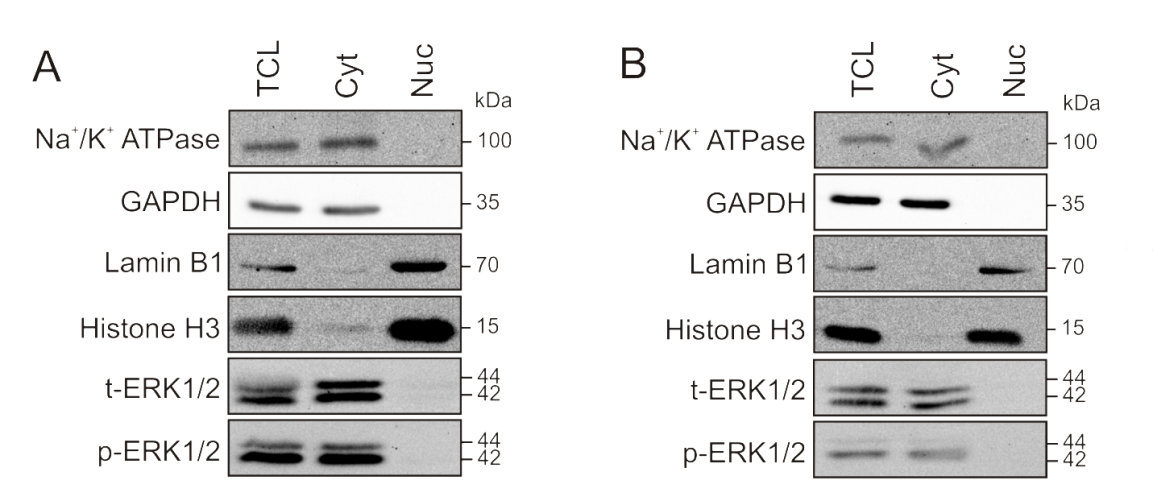


**Figure S3: Subcellular distribution of ERK1/2.** Another clone of hiPSCs (A) and NT2 cells (B) were used as pluripotent stem cells. Both cells showed the same pattern of dominant cytoplasmic localization of ERK1/2 and p-EKR1/2.


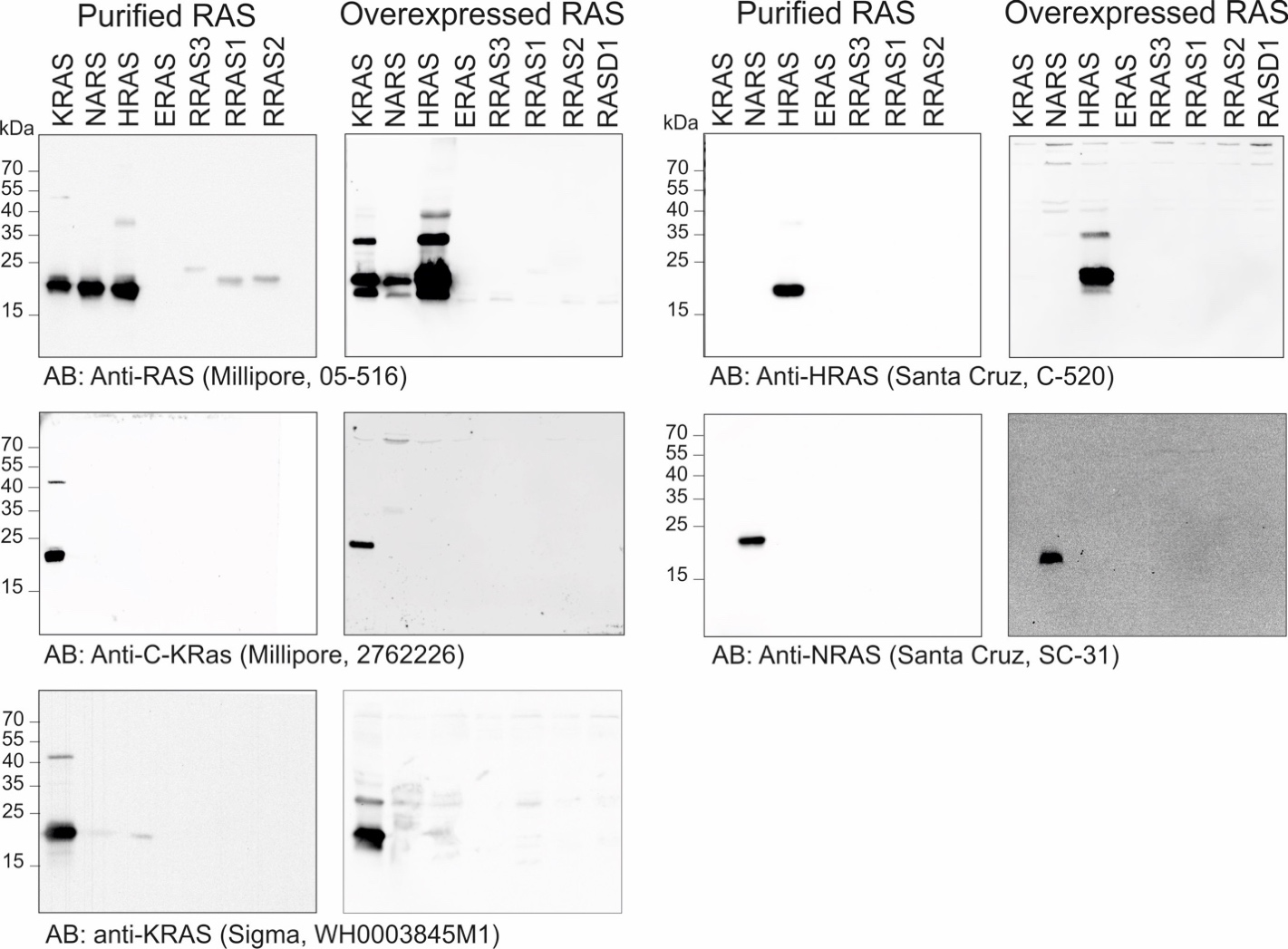


**Figure S4: Specification and validation of RAS antibodies.** Purified RAS proteins and overexpressed RAS in HEK cells were used for validation of different antibodies. Mouse anti-pan-RAS (Millipore, #05-516), mouse anti-KRAS (Sigma, WH0003845M1), mouse anti-NRAS (Santa Cruz, sc-31) and rabbit anti-HRAS (Santa Cruz, sc-520) were used as validated antibodies which could only recognize three canonical RAS paralogs, KRAS, NRAS and HRAS, respectively.
